# Short‐term meditation training influences brain energy metabolism: A pilot study on ^31^P MR spectroscopy

**DOI:** 10.1002/brb3.1914

**Published:** 2020-12-10

**Authors:** Elke R. Gizewski, Ruth Steiger, Michaela Waibel, Sergiy Pereverzyev, Patrick J. D. Sommer, Christian Siedentopf, Astrid E. Grams, Lukas Lenhart, Nicolas Singewald

**Affiliations:** ^1^ Department of Neuroradiology Medical University Innsbruck Innsbruck Austria; ^2^ Neuroimaging Research Core Facility Medical University Innsbruck Innsbruck Austria; ^3^ Yogamood Innsbruck Austria; ^4^ Center for Molecular Biosciences Innsbruck (CMBI) Department of Pharmacology and Toxicology Leopold Franzens University Innsbruck Austria

**Keywords:** brain imaging, fMRI, meditation, MR spectroscopy

## Abstract

**Background:**

Meditation is increasingly attracting interest among neuroimaging researchers for its relevance as a cognitive enhancement technique and several cross‐sectional studies have indicated cerebral changes. This longitudinal study applied a distinct and standardized meditative technique with a group of volunteers in a short‐term training program to analyze brain metabolic changes.

**Methods:**

The effect of 7 weeks of meditation exercises (focused attention meditation, FAM) was assessed on 27 healthy volunteers. Changes in cerebral energy metabolism were investigated using ^31^P‐MR spectroscopy. Metabolite ratios were compared before (T1) and after training (T2). Additional questionnaire assessments were included.

**Results:**

The participants performed FAM daily. Depression and anxiety scores revealed a lower level of state anxiety at T2 compared to T1. From T1 to T2, energy metabolism ratios showed the following differences: PCr/ATP increased right occipitally; Pi/ATP decreased bilaterally in the basal ganglia and temporal lobe on the right; PCr/Pi increased in occipital lobe bilaterally, in the basal ganglia and in the temporal lobe on the right side. The pH decreased temporal on the left side and frontal in the right side. The observed changes in the temporal areas and basal ganglia may be interpreted as a higher energetic state, whereas the frontal and occipital areas showed changes that may be related to a down‐regulation in ATP turnover, energy state, and oxidative capacity.

**Conclusions:**

The results of the current study indicate for the first time in a longitudinal study that even short‐term training in FAM may have considerable effects on brain energy state with different local energy management in specific brain regions. Especially higher energetic state in basal ganglia may represent altered function in their central role in complex cerebral distributed networks including frontal and temporal areas. Further studies including different forms of relaxation techniques should be performed for more specific and reliable insights.

## Graphical abstract

Within this MR spectroscopy study, we assessed the effect of 7 weeks of meditation exercises (focused attention meditation, FAM) from 27 healthy volunteers regarding brain metabolic changes. Our results indicate for the first time in a longitudinal study that even short‐term training in FAM has considerable effects on brain energy state in different brain regions.

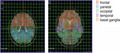

## 1 INTRODUCTION

There is evidence that meditation influences affective regulation and can reduce negative affect (Travis et al., 2018). It has also been shown to influence cognition and perception as well as physiological processes. Several studies have addressed physiological (see Ooi et al. (Ooi et al., 2017) for a review) and cerebral processing in experienced and/or non‐experienced meditation practitioners. The results show overall reduced alertness as well as a shift to vagal dominance. However, results have depended not only on meditation experience but also on the technique used. Focused attention meditation (FAM) has been associated with increased gamma electroencephalography (EEG) power and coherence (Braboszcz et al., 2017), and open monitoring meditation (OMM) with increases in theta, alpha, and beta bands (Ahani et al., 2013). Self‐transcending meditation has been described as related to alpha EEG bands (Travis et al., 2010).

The rapidly progressing science of meditation has led to insights into the neural correlates of different forms of meditation (FAM, OMM, compassion meditation [CM] and loving kindness meditation [LKM]) in regard to states and traits. In most techniques, attention is an important part of the process of meditation. Traditional yoga teaching describes two stages of meditation that occur in sequence: meditative focusing (*dharana*) and effortless meditation (*dhyana*) (Boccia et al., 2015). Especially the first step, meditative focusing, is a well‐described form of FAM that leads to effortless meditation. This mediation method can thus be used as a straightforward method to teach FAM in an experimental setting. Therefore, the intention of our study was to evaluate FAM. As an introduction to possible influences of FAM on cerebral processes, we provide an overview of published results dealing with this type of meditative practice to identify potential influences on cerebral processes.

With Resting state fMRI (rsfMRI), an influence of meditation on functional connectivity between affective networks and the posterior cingulate (PCC) and precuneus has been described—specifically, in regions involved in self‐referential processing was analyzed. The extent of this influence has depended on the different meditative stages (Baerentsen et al., 2010). Default mode network (DMN) functional connectivity and visual network analyses are presented by Berkovich‐Ohana et al. (2016), showing that connectivity within both networks was lower in meditators and correlated with meditative experience. Especially in mindfulness meditation and FAM, deactivation of DMN has been described, which underscores the theory that these techniques are goal‐oriented and result in the directing of attention (Brewer et al., 2011; Simon & Engstrom, 2015).

An extensive review of fMRI and PET studies with meditators provides an overview of various meditative techniques and their influence on brain activation patterns (Fox et al., 2016). Even though there are some brain areas that are recruited consistently across multiple techniques, including insula, pre/SMA, dorsal ACC, and the frontopolar cortex, a convergence of these areas, that is, recruitment of the same areas for all of the meditation techniques investigated, is described by the authors as the exception. This suggests that studies should choose meditation techniques carefully and ensure that they are performed in a standardized manner for all participants. Longitudinal studies should thus be helpful in overcoming such confounding variables. This underscores the choice of FAM in our longitudinal study as a structured, focused method that leads to comparable results within a group of participants.

To date, however, longitudinal studies addressing the effects of meditation on cerebral processing are rare. One recent study reported effects of a 4‐week Sahaja Yoga meditation training program on GM density and spontaneous resting‐state brain activity in a group of 12 meditation‐naïve healthy adults (Dodich et al., 2019). The authors showed that meditators had an increase in GM and changes in brain activation coherence in the right inferior frontal gyrus. A second study, with a longitudinal design and graph‐based analysis of rsfMRI, included elderly participants taking part in meditation training versus relaxation training for 8 weeks. The results showed that meditation training led to decreased intra‐connectivity in the DMN, salience network and SMN modules post‐training and decreased connectivity strength between the DMN and other modules. At a local level, meditation training lowered nodal strength in the left PCC, bilateral paracentral lobule and middle cingulate gyrus. The authors conclude that these changes represent a movement toward a more self‐detached perspective as well as more efficient cerebral processing. The results also support the hypothesis that short‐term meditation may be a beneficial method of mental training for the elderly (Cotier et al., 2017).

So far, capturing the BOLD effect (fMRI) has played a central role in measuring brain function, which is an indirect way to measure energy expenditure of the human brain. A more direct approach is the dynamic measurement of tissue concentrations of high‐energy phosphates as done in the current study. Resting state fMRI (rsfMRI) may be the method closest to the ^31^P‐MRS approach. MRS studies of meditative state are rare and have thus far been conducted only with ^1^H‐MRS which does not measure the energetic state of the brain.

^31^P‐MRS is a particularly promising MRS method in this context as it allows the measurement of cerebral energy metabolism in vivo. Some authors have even used this method for functional MRI of the visual cortex to indicate the connection between cerebral function and 31P MRS (Chen et al., 1997). To date, there have been no studies using phosphorous MR spectroscopy (^31^P‐MRS) under meditation. The unique advantage of ^31^P‐MRS is that it allows the measurement of various metabolites of energy metabolism and membrane turnover: adenosine triphosphate (ATP), phosphocreatine (PCr), inorganic phosphate (Pi) as well as various phosphomonoesters (PME) and phosphodiesters (PDE) (Chaumeil et al., 2009; Hugg et al., 1992; Wijnen et al., 2010). Direct quantification of metabolite concentrations from ^31^P spectra is complicated due to factors such as coil sensitivity, field inhomogeneity, and relaxation time.

However, some metabolite ratios have been shown to offer relevant and stable results and may therefore be seen as established ratios for the interpretation of energetic changes in cerebral areas. Changes in phosphorylation—indicated by Pi/ATP ratio—have been described as a marker of brain bioenergetics (D'Rozario et al., 2018). The PCr/ATP ratio has been interpreted as a marker of energetic state and phosphorylation potential. In ATP deficiency, creatine kinase equilibrium buffers ATP, resulting in a decrease of PCr and an increase of Pi and free creatine. Therefore, the Pi/ATP ratio may be interpreted as a marker of the amount of ATP turnover.

Furthermore, brain pH can be measured using ^31^P‐MRS by calculating the chemical shift difference between PCr and Pi (Cichocka et al., 2015; Petroff & Prichard, 1983). Normal brain activity can be associated with task‐related pH changes, as can environmental factors such as altitude (Shi et al., 2014). Therefore, ^31^P‐MRS seems to be an interesting additional method (to fMRI or volume‐based analysis) to identify changes in cerebral processes under meditative training and to provide additional insights into results from fMRI while direct measurement of energy metabolism.

Even short‐term meditative practice seems to affect brain activation and connectivity in rsfMRI studies, which led to our hypothesis that meditation should also affect brain energy metabolism in a longitudinal study. As changes in energy metabolites represent a form of functional information of the brain without direct dependency on blood oxygenation as in fMRI, MRS can be an addition to the already reported functional changes due to meditation. However, it is essential to bear in mind that individual mental state varies over time and throughout the day. Therefore, paradigms and settings used to study meditative effects on the brain should be designed carefully in order to exclude confounding factors as much as possible.

## 2 METHODS

### 2.1 Volunteers, inclusion, and exclusion criteria

Eighteen healthy females and twelve males were recruited by advertisement at a local yoga school. General exclusion criteria included age < 18 years, any concurrent medical condition, including neurological, psychiatric, cardiovascular, immunological and endocrine conditions, evidence of structural brain abnormality upon structural MRI scan, and the usual MRI‐specific exclusion criteria (i.e., phobic anxiety, claustrophobia, ferromagnetic implants). All participants had to commit to perform meditation regularly during the whole study period and to avoid alcohol, nicotine and coffee at least 4 hr before scanning. The study was conducted in accordance with the Declaration of Helsinki, and the study protocol was approved by the local Ethics Committee. All participants gave written informed consent.

### 2.2 Study design

This MRI study on healthy volunteers was designed as a longitudinal investigation. Participants were measured twice: the first time before beginning the meditation training process (T1) and the second time after 7 weeks of regular meditation practice as described below (T2). During this time, participants were required to attend 14 instruction sessions. Additionally, an app was used to support the learning and training process. On study day T1, participants first filled out a short questionnaire. Second, a structural MRI scan was taken to exclude brain abnormalities and to familiarize subjects with the scanning environment. Third, ^31^P‐MRS was performed during “meditative” state, meaning that each participant was in a relaxed state, focusing on breathing. On study day T2, participants again filled out a short questionnaire. ^31^P‐MRS was performed during “meditative” state (now using the learned meditative technique). All volunteers were scanned during the evening to account for changes throughout the day.

### 2.3 Questionnaires and subjective ratings

To evaluate possible interference due to the scanning environment, participants completed ratings before and after scanning using visual analogue scales (VAS) on meditation depth: 0 meaning no meditative state reached, 10 meaning deepest meditative state ever reached. Additionally, at the beginning and end of the study, participants completed questionnaires about their lifestyle and medical history (SF‐36, V 1.0, adopted from (Ware & Sherbourne, 1992)), the Handedness Questionnaire (the Edinburgh Inventory) and the State‐Trait‐Angst‐Depressions‐Inventar (STADI) (Renner et al., 2018).

The German version of the STADI is a 40‐item questionnaire based on self‐reporting. This psychological inventory measures current state of anxiety (S‐anxiety), including agitation, apprehension, euthymia and dysthymia, as well as the relatively stable aspects of trait anxiety, that is, personal characteristics (T‐anxiety), general states of calmness, confidence, and security (Julian, 2011). The responses are given on a 4‐point Likert‐type scale for S‐anxiety (1 = not at all, 4 = very much so) and for T‐anxiety (1 = almost never, 4 = almost always). This questionnaire was filled out at baseline and at follow‐up by each participant.

### 2.4 Meditative technique and training protocol

After the first MRI appointment (see above), all enrolled participants attended 14 sessions of guided meditation over 7 weeks. The focus of this meditation technique, which is based on Raja Yoga, is, ultimately, to completely stop thinking. All sessions lasted 45 min and were led by one of the authors (MW), an academic instructor with over 15 years of experience in teaching yoga and a 4000‐hr certification as a yoga teacher. Classes were offered 2 times per week to enable participants to fulfill the program requirements. Participants were asked to perform home‐based practice daily for at least 15 min throughout the study period.

In the first 4 sessions, participants were instructed in certain elements of breathing *(pranayama*) and retraction of the senses *(pratyahara*). The students learned, for example, how to blend out environmental stimuli and focus instead on their own body, concentrating their mind (*dharana*). These steps are considered preliminary stages of meditation (*dhyana*). Specifically, participants practiced *ujjayi* breathing, which entailed lying down on a cushion, expanding the rib cage and learning to feel their lungs, lengthening inhalation, slowing down exhalation, and allowing the breath to become smooth and even. Inhaling through both nostrils on 4 counts and exhaling through both nostrils on 8 counts is called *ujaii visama vritti*. Participants used this breathing technique to calm the mind. During the second part of meditation sessions, participants were asked to sit in an upright position, finding the right support using various props: sitting on a block, cushion or blanket in a special meditation position they could choose, either cross‐legged *(siddhasana)* or kneeling *(virasana)*. A special technique of alternating air breathing, called the *nadi shodhana pranayama* technique, was explained and practiced. The technique began with inhaling through both nostrils, closing the right nostril and exhaling through the left nostril, then inhaling through the left nostril and exhaling through the right nostril. Participants’ sitting position was aligned using visualization of a mountain: “Feel your legs from the toes to the hip joints, and feel them heavy and still. Still as a mountain. Nothing can move them...” This technique was continued for all parts of the body, leading to a statue‐like body feeling, still and upright. In the ensuing 15‐min meditation, participants used visualization of a mountain and the mantra: “upright and still.” For at‐home practice, participants used the same visualization technique, supported by a CD that was created for the purposes of this study.

For the 5th to 9th sessions, participants began in their seated position and practiced *nadi shodhana pranayama* for 8 min. and breath observation for 2 min. This was followed by 25 min. of guided meditation, entailing observing the mind and calming one's thoughts to achieve inner silence. This meditation technique is called *antar mouna*.

In sessions 10–12, participants were instructed to practice 5 min. of *nadi shodhana* in seated position and then to assume a lying position. For 3 min., they then practiced silent breath observation, followed by deep relaxation in this lying position, also known as “yogic sleep” or *yoga nidra*.

In the 13th and 14th sessions, the same procedures as in sessions 10–12 were practiced, but now with simulation of the study conditions during an MRI session—including, in particular, exposure to the typical noise associated with an MRI session.

At the second MRI appointment, participants filled out the questionnaire and practiced *nadi shodhana pranayama* for 5 min. before they were placed into the MRI scanner. Once inside the scanner, they performed 3 min. of breath observation followed by *yoga nidra* in combination with *antar mouna* throughout the remaining scanning period.

### 2.5 Structural MRI

All MR images taken to exclude structural pathologies were acquired using a 3 T MR system (Skyra, Siemens Medical AG, Erlangen, Germany), with a standard 64‐channel head coil. Structural MRI included a fluid‐attenuated inversion recovery sequence (FLAIR) with a voxel size of 0.9 × 0.9 × 3.0 mm^3^ (TR = 10,000 ms, TE = 90.0 ms, TA = 4:42), a coronal‐oriented, magnetization‐prepared rapid gradient echo (MPRAGE) with 1.0 mm isotropic resolution and an acquisition time (TA) of 5:47 (TR = 1,800 ms,TE = 2.22 ms), and a transverse‐oriented, diffusion‐weighted sequence (DWI) with an acquisition time of 5:28 and a voxel size of 2 × 2 × 2 mm^3^ (TR = 9,600 ms, TE 92 ms). When no meditative state was instructed, subjects were asked to lie still and relaxed with their eyes closed during the acquisition process.

### 2.6 ^31^P‐MRS and analyses

^31^P‐MRS was performed on a 3T whole‐body, multi‐nuclear system (Skyra, Siemens Medical AG, Erlangen, Germany) with a double‐tuned ^1^H/^31^P volume head coil (Rapid Biomedical, Würzburg, Germany). For each volunteer, one MRS three‐dimensional block of the whole brain was recorded. For planning, a T2‐SPACE sequence was acquired (sagittal‐oriented, T2‐weighted 3D sequence with isotropic resolution and a voxel size of 1.2 × 1.2 × 1.2 mm^3^ (TR = 3,000 ms, TE = 412.0 ms, TA = 2:50)). In order to achieve as much volume of the brain as possible, the coronal inclination was aligned on the dorsal line of the brain stem and axial arrangement with as little inclusion as possible of cavities and skull and avoidance of fat, bone, and boundary layers perturbing the ^31^P spectra. The volume of interest was obtained with an extrapolated 8 × 8 × 8 matrix and a field of view of 240 × 240 × 200 mm^3^, resulting in a voxel size of 15 × 15 × 25 mm^3^. Similar to the process described by Hattingen et al. (Hattingen et al., 2009), we performed MRS acquisition with WALTZ 4 proton decoupling, repetition time TR 2000 ms, echo time TE 2.3 ms, flip angle 60°, and 10 acquisitions for averaging.

### 2.7 Data processing and analysis

^31^P‐MRS data were processed offline with the jMRUI software package (version 5.0, http://www.mrui.uab.es) utilizing the non‐linear least square fitting algorithm AMARES, which considers prior knowledge (Vanhamme et al., 1997). The fitting model was composed of 12 Lorentzian‐shaped, exponentially decaying sinusoids as follows: phosphocholine, phosphoethanolamine (the sum of both referred to as phosphomonoesters or PME), inorganic phosphate (Pi), glycerophosphocholine, glycerophosphoethanolamine (the sum of both referred to as phosphodiesters or PDE), phosphocreatine (PCr) and adenosine triphosphate (ATP) consisting of two doublets (γ‐ATP and α‐ATP) and triplets (β‐ATP), which were added together. The peak of β‐ATP can be used as an internal quantification as it is considered uncontaminated by α‐ADP, nicotinamide adenine dinucleotide NAD and NADH (oxidized) contributions to α‐ATP (Du et al., 2007). However, we calculated the mean of α‐ATP, β–ATP, and γ‐ATP as our reference value and designated it ATP. Intracellular pH was calculated using the chemical shift of Pi relative to PCr, using the formula of Petroff and Prichard (1983). Absolute quantification of metabolite concentrations from 31P spectra was found to be inefficient within that study due to factors such as coil sensitivity, field inhomogeneity, and scanning time. It has since been shown that using metabolite concentration ratios in order to evaluate metabolic changes is more stable (Liu et al. 2017).

**Figure 1:**
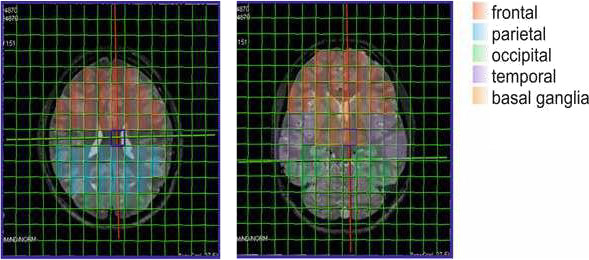
Two exemplary layers of the ^31^P‐MRS acquisition grid co‐registered on the axial T2‐SPACE sequence are shown, with volumes of interest detailed in color

The brain areas (frontal, parietal, occipital, temporal, and basal ganglia) were delineated by two experienced neuroradiologists (CS, AEG) and analyzed with one or more ^31^P‐MRS voxels in each volunteer (see Figure 1). The amount of the included voxels per area depended on the size of the respective area and the quality of the spectra. CSF was excluded, and due to the large voxel size of 1.5 × 1.5 × 2.5 mm^3^, only voxels which contained at least 2/3 of the targeted ROI were incorporated. However, within the voxels gray and white matter could not be distinguished. Each single spectrum was assessed visually according to the criteria set forth by Kreis (Kreis, 2004), and metabolite ratios were calculated afterward. The ratios calculated were PCr/ATP, PCr/Pi, and Pi/ATP. Additionally, pH values were calculated. Statistical analysis of ratios was performed using Graphpad Prism version 8 (Graphpad Inc.). Data normality of metabolite ratios was assessed with the one‐sample Kolmogorov–Smirnov test at a 5% significance level. Outliers were identified with the ROUT method. The metabolite data were not normally distributed, and a Mann–Whitney *U* test was therefore applied for the investigation of differences between groups. In order to address the problem of multiple comparisons, the Bonferroni method was applied. Therefore, *p*‐values < 0.00125 were considered statistically significant. Analysis of the correlation between VAS rating of meditative state as well as the amount of daily meditative practice and ^31^P metabolite ratios was done using Spearman's correlation coefficient, with *p*‐values < 0.01 considered statistically significant.

### 2.8 Statistical analysis of ratings and questionnaires

VAS ratings were analyzed with Student's *t* test. A paired *t* test was performed to assess in‐between group differences of the STADI and SF‐36 from T1 and T2. In all analyses, the alpha level for significance was set at 0.05, and results are shown as mean ± standard error of the mean (*SEM*).

## 3 RESULTS

### 3.1 Participants

A total of 27 healthy subjects participated (*n* = 10 male; *n* = 17 female) as 2 males and one female did not fulfill the inclusion criteria. Full data sets were available for *n* = 27 subjects. The mean age was 42.67 years (standard deviation 13.01; range: 22–69 years).

### 3.2 Behavioral results

All included participants (27) were able to attend the meditation courses regularly and performed regular self‐practice 5–7 times per week. The range was similar in both gender groups. Within the whole group, 25 participants practiced daily, one 6 times per week, and one 5 times per week. During this self‐practice, the average duration was 24 min per day (range: 15–45 min).

All participants except one also performed athletic activities (6–7 times per week, total duration between 50 min and 3 hr) including yoga, jogging, skiing, biking, hiking, climbing, golfing, and swimming. None had previous experience in meditative practice. One person did walking sessions 5 times per week. There was only one female who smoked regularly during the study period; alcohol use was seldom within the whole group (ranging from 0 to 3 times per week, 1–2 glasses of wine or beer). In terms of eating behavior, most participants were omnivorous, with four vegetarians (1 male and 3 females) and one pescatarian. There were no lifestyle changes during the 7 weeks of meditation training. All but one of the participants were right‐handed.

One female had a history of chronic lymphatic leukemia in full remission; no other severe diseases were reported by the participants.

Trait anxiety and depression scores, assessed with the STADI, were within normal ranges. The comparison of T1 and T2 revealed significant differences only in s‐STADI (state anxiety), with a higher anxiety level at T1. Additionally, ratings in the sub‐item dysthymia improved at T2 (*p* > .05). In SF‐36 analysis, we found only one significant change at *p* < .05 in the body‐related items but an improvement from T1 to T2 in the psychological items. The relevant results are summarized in Table 1.

**Table 1:** Group characteristics and main results of the questionnaires

		T1	T2	
Age (years)	42.7 (IQR = 21)			
gender (m/f)	10/ 17			
SF‐36 score bodily pain		56.69 ± 5.77	55.69 ± 6.28	NS
SF‐36 score mental health		47.69 ± 9.47	52.08 ± 7.77	***p = .0148***
STADI‐S score anxiety		43.99 ± 14.87	34.26 ± 7.58	***p = .0027***
STADI‐S score depression		33.18 ± 13.35	34.26 ± 9.67	NS
STADI‐T score anxiety		45.27 ± 11.51	44.11 ± 9.93	NS
STADI‐T score depression		40.88 ± 8.36	40.63 ± 8.65	NS
STADI‐S sub‐score dysthymia		47.35 ± 8.44	43.56 ± 8.12.5	***p = .0291***
depth meditative state MRI		na	6.41 ± 1.45	

Note

T1: time point 1: before meditative training, T2: time point 2 (after learning meditation): after 7 weeks of meditative training. SF‐36: Short Form 36 Health Survey, STADI: State‐Trait‐Angst‐Depressions‐Inventar.

*P* values  <  .05 are considered statistically significant and indicated in bold.

All volunteers were able to achieve a meditative state in the scanner environment. The average subjective rating of the depth of meditative state during MRI scanning at T2 was 6.41 ± 1.45 (females: 6.35, males: 6.5, no significant difference) in a VAS from 0 to 10; the range was 4–10. A total of twenty participants achieved a meditative depth ≥ 6, considered excellent performance in meditation. All ratings are summarized in Table 1.

#### 3.2.1 ^31^P‐MRS

Mann–Whitney *U* tests of T1 and T2 showed differences in energy metabolism ratios in several brain areas, predominately on the right side.

**Figure 2:**
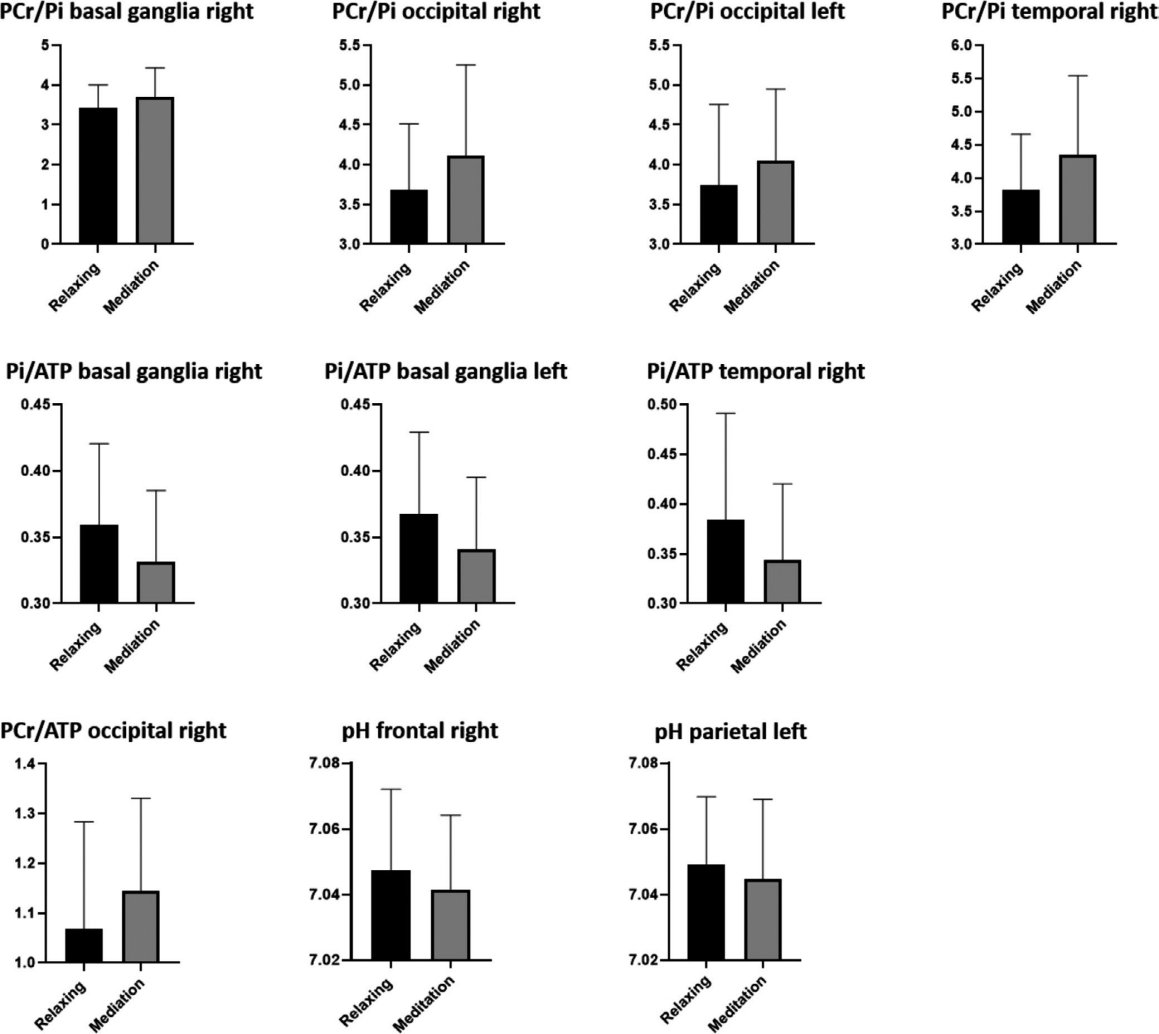
Bar graphs with mean values and standard deviations of all significant differences (*p* < .00125) between T1 and T2

The PCr/ATP ratio increased in the occipital regions from T1 to T2 on the right hemisphere. Pi/ATP ratio decreased bilaterally in the basal ganglia and in the temporal region on the right side. The PCr/Pi ratio increased in the occipital regions bilaterally and in the basal ganglia and temporal regions on the right side. A decrease in pH was found in the frontal region on the right side and in the parietal regions on the left side. All other comparisons showed stable ratios and values between T1 and T2. Detailed data are given in Table 2. Figure 2 shows the diagrams of the significant changes for each region and ratio. No significant correlation was found between depth of meditative state and any metabolite ratio.

**Table 2:** Mean values and standard deviations (in parentheses) of ^31^P‐MRS comparing T1 relaxation and T2 meditation. Significant differences are shown in bold

	right			left			
PCr/ATP				PCr/ATP			
Region	T1	T2		Region	T1	T2	
basal ganglia	1.205 (0.129)	1.190 (0.130)	NS	basal ganglia	1.211 (0.129)	1.201 (0.140)	NS
frontal	1.163 (0.209)	1.173 (0.217)	NS	frontal	1.115 (0.199)	1.163 (0.214)	NS
occipital	**1.068 (0.216)**	**1.144 (0.187)**	***p* = .0004**	occipital	1.085 (0.226)	1.140 (0.171)	*NS*
parietal	1.060 (0.127)	1,065 (0.125)	NS	parietal	1.056 (0.110)	1.059 (0.110)	NS
temporal	1.401 (0.201)	1.429 (0.233)	NS	temporal	1.405 (0.201)	1.403 (0.226)	NS
Pi/ATP				Pi/ATP			
Region	T1	T2		Region	T1	T2	
basal ganglia	**0.360 (0.061)**	**0.331 (0.054)**	***p* < .0001**	basal ganglia	**0.36 (0.062)6**	**0.341 (0.054)**	***p* < .0001**
frontal	0.346 (0.100)	0.334 (0.079)	NS	frontal	0.349 (0.076)	0.353 (0.094)	NS
occipital	0.297 (0.067)	0.291 0.067)	NS	occipital	0.298 (0.066)	0.290 (0.056)	NS
parietal	0.322 (0.066)	0.321 (0.063)	NS	parietal	0.309 (0.055)	0.310 (0.066)	NS
temporal	**0.384 (0.107)**	**0.344 (0.076)**	***p = *.0005**	temporal	0.373 (0.087)	0.378 (0.104)	NS
PCr/Pi				PCr/Pi			
Region	T1	T2		Region	T1	T2	
basal ganglia	**3.424 (0.582)**	**3.692 (0.742)**	***p* = .0005**	basal ganglia	3.369 (0.587)	3.600 (0.675)	NS
frontal	3.539 (0.926)	3.641 (0.869)	NS	frontal	3.310 (0.769)	3.414 (0.700)	NS
occipital	**3.685 (0.828)**	**4.113 (1.140)**	***p* < .0001**	occipital	**3.746 (1.012)**	**4.054 (0.897)**	***p* = .0002**
parietal	3.408 (0.765)	3.429 (0.698)	NS	parietal	3,513 (0.611)	3,561 (0,857)	NS
temporal	**3.824 (0.837)**	**4.347 (1.193)**	***p* < .0001**	temporal	3.931 (0.970)	3.887 (0,869)	NS
PH				PH			
Region	T1	T2		Region	T1	T2	
basal ganglia	7.044 (0.019)	7.039 (0,020)	NS	basal ganglia	7.045 (0.020)	7.042 (0.020)	NS
frontal	**7.049 (0.027)**	**7.042 (0.029)**	***p* < .0001**	frontal	7.044 (0.027)	7.042 (0.026)	NS
occipital	7.054 (0.023)	7.054 (0.029)	NS	occipital	7.057 (0.030)	7.054 (0.022)	NS
parietal	7.048 (0.020)	7.046 (0.018)	NS	parietal	**7.049 (0.021)**	**7.045 (0.024)**	***p* < .0001**
temporal	7.067 (0.033)	7.058 (0.034)	NS	temporal	7.058 (0.024)	7.057 (0.030)	NS

Note

PCr: phosphocreatine, ATP: adenosine triphosphate, Pi: inorganic phosphate, gray: non‐significant. *p* < .05 was used as the level of significance. NS: not significant.

In addition, we calculated the regional differences for all ratios (meaning the ratio differences between the brain regions at each time point) and found that nearly all metabolite ratios between the analyzed brain regions remained stable from T1 to T2. Only the Pi/ATP ratio changed from a significant difference between the basal ganglia and frontal lobe at T1 to a non‐significant one at T2.

Analysis of correlation between VAS rating of meditative state as well as amount of daily meditative practice and ^31^P metabolite ratios revealed no significant results.

## 4 DISCUSSION

Our results suggest the influence of short‐term meditation training on focused attention processing and related brain energy metabolism in different brain areas. Various changes in ATP turnover, PCr buffering, and Pi regulation as well as in pH values were revealed. Three brain regions were particularly relevant for energy metabolism change during meditation: the basal ganglia and the temporal and occipital lobes. In detail, a stable PCr/ATP ratio and decrease in Pi/ATP ratio were found in the basal ganglia of both hemispheres; in addition, the PCr/Pi ratio increased on the right basal ganglia. PCr/ATP values increased in the occipital lobe of the right hemisphere. Moreover, PCr/Pi increased in the occipital lobe of both hemispheres. In the temporal lobe on the right side, a decrease in Pi/ATP values and an increase in PCr/Pi values—but stable PCr/ATP values—were found. Additionally, in the frontal lobe of the right and the parietal lobe of the left hemisphere, decreasing pH values were identified.

The PCr/Pi ratio has been shown to correlate positively with phosphorylation potential or tissue oxygenation and can be interpreted as a marker of metabolic oxidative capacity and energy reserve (Chance et al., 2006). Changes in the steady state levels of energetic phosphates (PCr and Pi) are not expected to vary significantly in the healthy brain. Instead, oxidative metabolism should dominate in the brain to provide ATP (Andres et al., 2008; Chen et al., 1997; Kemp, 2000; Zhu et al., 2012). However, the ratio changes found in the basal ganglia and temporal area indicate a slight decrease in Pi and increase in ATP. This fits well with the decrease of Pi/ATP values in both areas.

Therefore, the basal ganglia and right temporal regions seem to be more active in a learned meditative state during focused attention. It has been suggested that, under conditions of activation, a transient inadequacy and delay in the supply of oxygen and glucose occur, leading to a transient uncoupling of glycolysis and the Krebs cycle (Dienel & Hertz, 2001; Lowry & Fillenz, 1997). Rea et al. state that it is during this period of transient metabolic alteration that the local ATP buffering capacity and ability to rapidly resynthesize ATP may be of importance for the performance of cognitive tasks (Rae et al., 2003). A higher oxidative capacity can also be assumed for the basal ganglia of the right hemisphere due to meditation, which is indicated by the highly significant elevated PCr/Pi ratio. In these regions, the Pi/ATP ratio also decreases significantly from T1 to T2, which can be interpreted as an increase in ATP turnover in the meditative state compared to individual relaxation with focus on breathing. Thus, a decrease of Pi through meditation can be assumed. Normally, alteration of Pi is related to changes in ATP to ensure that ATP hydrolysis remains unchanged. In a healthy brain, energy metabolism is known to be in a well‐defined equilibrium (Ames, 2000).

In the occipital region, however, we found a different change in metabolite ratios. The PCr/ATP ratio increased in the occipital lobe of the right hemisphere after performing the meditation technique. ATP constitutes a direct energy source for energy‐consuming processes within cells, whereas PCr acts as an energy storage compound. Therefore, the PCr/ATP ratio has been interpreted as a marker of energetic state (Beer et al., 2002; Kemp, 2000). One study with intensive training on a cognitive task showed an increase in the total pool size of PCr (Valenzuela et al., 2003). Furthermore, changes in cerebral pH due to cognitive task performance have been shown in male volunteers (Rae et al., 2003). In that study, correlations in both adults and children between Pi‐containing peak ratios (Pi/ATP) and verbal cognitive tasks were found. In our study, the results after learning meditation might be interpreted as an increase in PCr (and perhaps a decrease in ATP and Pi in a stable relationship, given that Pi/ATP values did not change), which may indicate a deactivation of the occipital area. Moreover, the increase in the PCr/Pi ratio in both occipital lobes can be interpreted as indicative of a lower energetic state and higher oxidative capacity in this area, induced by a meditative state. (Vink et al., 1987; Welch et al., 1989). One study analyzing resting wakefulness found a correlation between Pi/ATP ratio and lower vigilance levels, meaning that an increase in this ratio reflects a decrease in phosphorylation potential (D'Rozario et al., 2018). In our study, no ratio changes were found in the parietal regions, where only the pH value decreased slightly from T1 to T2 in left hemisphere. This decrease can be interpreted as a reduced ratio of oxidative phosphorylation to glycolysis (Chouinard et al., 2017). In normal aging, pH has been found to decrease in brain tissue (Mandal et al., 2012). However, pH reduction and a decrease in PCr/Pi ratio have also been described during brain stimulation (Chen et al., 2018). As the changes in our group are minimal (yet significant) and the PCr/Pi ratio shows different behavior in the basal ganglia and occipital and temporal regions, the relevance of these changes remains unclear. Normally, energy metabolism of the brain and intracellular pH levels is controlled chiefly through a buffer process of the CO_2_/HCO_3_ system, which would explain only small changes in pH detectable in ^31^P‐MRS (Hendriks et al., 2019).

In addition, calculations of regional differences for all ratios compared to each region revealed that nearly all metabolite ratios were stable from T1 to T2. Therefore, the overall distribution of energetic compounds remained unchanged. Only the Pi/ATP ratio changed to a non‐significant difference between the basal ganglia and frontal lobe at T2 (which was significant at T1), which might suggest an altered connectivity between the frontal cortex and basal ganglia. Some rsfMRI studies also reported changes in connectivity between frontal areas and basal ganglia (Hernandez et al., 2018).

In summary, the basal ganglia appears to be a key region in meditative state, and the Pi/ATP ratio represents an important parameter in vigilance and cognitive processing. How should this be interpreted in relation to other functional studies? Several fMRI studies suggest mechanisms for the various forms of meditation, with the down‐regulation of brain network activities being pointed out for FAM (Fox et al., 2016). In OMM, the gating and tuning of brain network coupling have been described. For most meditation forms, asymmetry to the left has been described in top–down regulation and enhanced inter‐hemispheric integration in meditation states. Therefore, meditation has been proposed as providing a meta‐function for efficient brain/mind regulation (Raffone et al., 2019). The changes in energetic states in the occipital regions can be seen as in line with the published down‐regulation in rsfMRI during FAM (Raffone et al., 2019). However, our results are partly contradictory, given that more effects were seen in the right hemisphere and no reduction in energetic state in the parietal areas, which would be expected.

As there were no previous findings for ^31^P‐MRS in meditative states, we included published results dealing with rsfMRI in our hypothesis. Our hypothesis, that meditation would also effect brain energy metabolism, was supported, at least preliminarily, by our study. In fMRI meditation literature, several key regions have been described as activated in meditative states: prefrontal cortex, ACC, amygdala, and hippocampus (Jindal et al., 2013). As the resolution of ^31^P‐MRS is lower and not comparable with fMRI regions, these areas cannot be related directly to our ^31^P‐MRS results. Further recent fMRI studies have also shown decreased activation in the DMN (ACC, fusiform gyrus, middle temporal gyrus, precuneus) in comparison to an active task (Garrison et al., 2015; Tomasino et al., 2012). The decrease in ATP found in our study can be seen as in line with the deactivation of the DMN—even though not all areas showed a decrease in ATP: The temporal region, for example, can be interpreted as being more active in our study. However, neural correlates for just one meditation method, that is FAM, have yielded contradictory results. Therefore, it might be helpful to distinguish between internal and external attention as well as between different phases within one meditation technique (Scheibner et al., 2017). This was one reason we chose to compare meditation in T2 with focused attention on breathing before learning meditation and to use a longitudinal approach.

Taken together, our study provides first evidence that 31P MRS can reveal energy metabolism changes due to meditation, which—at least in some key areas—is in line with other functional studies such as those dealing with rsfMRI.

The present study has several strengths. Longitudinal studies are rare, and no previous comparable study had investigated FAM. One study, however, analyzed DMN changes after 40 days of mindfulness meditation training in novices, focusing on potentially altered depression symptomatology and anxiety. The results also showed cerebral structural and functional effects (Yang et al., 2019). That study additionally revealed changes in trait anxiety. In our study, a reduction in STAID ratings was also seen in the state anxiety part. However, in the DMN study, the number of participants was smaller than in ours, with 4 males and 10 females. Our STAID results indicate that even a short‐term learning period for meditation with a well‐designed program (regular and well‐structured instruction, personal commitment to daily practice and app support) can influence trait anxiety levels.

Meditation can be viewed as a cognitive enhancement technique. One study used a Stroop Color‐Word Task (SCWT) MRI paradigm to measure the performance of meditators compared to non‐meditators before and after a Zen meditation retreat (Kozasa et al., 2018). They found no significant differences between meditators and non‐meditators in the number of correct responses before and after the retreat. However, looking at the contrast between incongruent and neutral trials, brain activity in meditators was lower compared to non‐meditators in the VMPF, ACC, caudate/putamen/pallidum, temporal lobe, insula/putamen/temporal lobe, and PCC before the retreat. It would thus be of great interest to repeat our study with long‐term meditators before and after such a retreat. As our study revealed differences after short‐term and—compared to a retreat—less intense meditation training, the results should be even more pronounced after more intense training.

Such an approach would also overcome one of the shortcomings of this first exploratory study, that is, the comparison between a “subjective” relaxation state with focused attention on breathing at T1 to “learned and standardized” meditation at T2. Moreover, the meditation technique used in this study, FAM, also has its potential shortcomings. As in many other techniques, FAM consists of several stages and foci. Traditional yoga texts describe two stages of meditation that follow each other in sequence: meditative focusing (*dharana*) and effortless meditation (*dhyana*). A review evaluating eight experimental studies conducted on healthy participants who practiced *dharana* and *dhyana* indicates that there are differences between *dharana* and *dhyana*, even though they are both meditative states (Telles et al., 2016). Changes in electrodermal activity, cutaneous blood flow, and heart and breath frequency suggested lower sympathetic activity and arousal during *dhyana* (Raghavendra & Telles, 2012). During *dhyana*, only experienced meditators showed significant activation in the right middle temporal cortex, right inferior frontal cortex, and left lateral orbital gyrus. Activation in these areas was understood as suggesting that *dhyana* is associated with sustained attention, memory, semantic cognition, and an increased ability to detach mentally. We cannot exclude that our participants experienced switching of state during the scanning period.

A potential further limitation of our study is that the scanning sequence followed a fixed order, and participants had to switch from one state to another within a few minutes. The only way to determine whether participants were able to do so was via their self‐reporting on VAS, that is, without any objective biological markers. A mitigating factor was that participants had been trained to perform *dharana* and *dhyana* in a simulated scanner environment 3 weeks prior to testing.

Another limiting factor of this study is its reliance on ^31^P‐MRS, which offers low spatial resolution compared to other imaging modalities such as PET and, especially, fMRI. As magnetic resonance spectra are collected from a large volume of brain, interpreting these data raises the question of whether the observed changes in pH or metabolite concentrations reflect underlying biochemical events or are instead a consequence of variation in cellular composition (Fitzpatrick et al., 1996; Gilboe et al., 1993, 1998). The analyzed regions also include different types of gray matter (basal ganglia and cortical areas) but also different types of white matter fiber tracts. For example, the temporal area contains less white matter and has connection mainly within the brain (e.g., cingulum bundle). The basal ganglia region includes many primary white matter fiber tracts and connecting fibers to subcortical and spinal tracts. Therefore, also the main orientation of the white matter tracts differs between the regions and may have influence in energy metabolism. We therefore focused our interpretation on the longitudinal changes and not on the different metabolite concentration between brain areas.

Furthermore, PCr/Pi can be inaccurate, given the difficulty of reliably measuring the small Pi peak in a potentially noisy baseline. Another point of criticism might be our use of metabolite concentration ratios, with internal referencing rather than absolute metabolite concentrations. Obtaining absolute metabolite concentrations using an external reference with known concentrations and matched electromagnetic properties is time‐consuming and can be confounded by introducing variability, for example, with differences in coil loading. We therefore decided to analyze metabolite ratios that are established markers of energetic processes in the brain.

An additional limitation is the considerable range of the age of the participants. It is likely that age influences the cerebral distribution of energy metabolites, the baseline status of energetic metabolism, and the ability of the brain to adapt to these processes during meditation practice. Due to the relatively small number of volunteers in this pilot study, influences from age or gender were not investigated. However, as longitudinal changes within individual people were the topic of the present study and each participant represented her or his own control, the above‐mentioned effects might play a less important role for the core results of the study.

## 5 CONCLUSIONS

The results of the current study indicate for the first time in a longitudinal study that even short‐term training in FAM may have considerable effects on brain energy state with different local energy management in specific brain regions. Especially higher energetic state in basal ganglia may represent altered function in their central role in complex cerebral distributed networks including frontal and temporal areas. Further studies including different forms of relaxation techniques should be performed for more specific and reliable insights.

## CONFLICT OF INTEREST

The authors declare no conflicts of interest.

## AUTHOR CONTRIBUTIONS

Elke R. Gizewski^1,2^ involved in substantial contributions to conception and design, and interpretation of data. Ruth Steiger^1,2^ involved in acquisition of data, substantial contributions to conception and design, and interpretation of data. Michaela Waibel^4^ involved in acquisition of data, substantial contributions to conception and design, and interpretation of data. Sergiy Pereverzyev Jr.^1,2^ involved in analysis. Patrick J.D. Sommer^1^ involved in analysis. Christian Siedentopf ^1,2^ involved in substantial contributions to conception and design, and interpretation of data. Astrid E. Grams^1,2^ involved in analysis and interpretation of data. Lukas Lenhart^1,2^ involved in analysis. Nicolas Singewald^3^ involved in substantial contributions to conception and design. Additionally, all authors were involved in drafting the manuscript and revising it critically for important intellectual content, and have given final approval of the version to be published.

### Peer Review

The peer review history for this article is available at https://publons.com/publon/10.1002/brb3.1914.

## Data Availability

Data available on request from the authors. The data that support the findings of this study are available from the corresponding author upon reasonable request.
